# Trends in Within-Class Changes in US Average Wholesale Prices for Brand-Name Medications for Common Conditions From 2015 to 2020

**DOI:** 10.1001/jamanetworkopen.2020.35064

**Published:** 2021-01-22

**Authors:** Patrick Liu, Sanket S. Dhruva, Nilay D. Shah, Joseph S. Ross

**Affiliations:** 1 Yale School of Medicine, New Haven, Connecticut; 2 Department of Medicine, School of Medicine, University of California, San Francisco; 3 San Francisco VA Medical Center, San Francisco, California; 4 Division of Health Care Policy and Research, Kern Center for the Science of Health Care Delivery, Mayo Clinic, Rochester, Minnesota; 5 Department of Internal Medicine, Yale School of Medicine, New Haven, Connecticut

## Abstract

This cross-sectional study examines within-class changes in US wholesale drug prices for brand-name medications from 2015 to 2020.

## Introduction

Several new medications for treating common chronic conditions have come to market in the US in recent years. While such increased competition might be expected to decrease drug prices, policies promoting competition among brand-name drugs within the same class have not been associated with lower list prices.^1^ In fact, an analysis of the wholesale acquisition costs of insulins from 2012 to 2016, when only branded formulations were available, demonstrated compound annual growth rates (CAGRs) in costs ranging from 15% to 17%.^2^ The objective of this cross-sectional study was to assess patterns in price changes for multiple brand-name medications within the same drug class existing in the US market contemporaneously.

## Methods

We conducted a cross-sectional study of per-pill average wholesale prices (AWPs) in the US from August 13, 2015, to August 13, 2020, obtained the Micromedex Red Book (IBM).^3^ The study did not require institutional review board approval or patient informed consent because it was based on publicly available information and involved no patient records. This study is reported following the Strengthening the Reporting of Observational Studies in Epidemiology (STROBE) reporting guideline.

We limited our study sample to brand-name medications used for chronic conditions available for purchase before January 1, 2018, to better characterize pricing trends for prescription medications used over a prolonged period. There were multiple brand-name medications on the market contemporaneously in the following classes: direct oral anticoagulants (DOACs), sodium-glucose transport protein-2 (SGLT2) inhibitors, dipeptidyl peptidase-4 (DPP4) inhibitors, glucagon-like peptide-1 (GLP-1) receptor agonists, and platelet P2Y_12_ inhibitors. For each medication, we selected the recommended maintenance dosage on the label, limiting to medications sold by the manufacturer that received initial Food and Drug Administration approval.

Our primary outcome was the correlation in AWP unit prices among the multiple brand-name medications within each class available over time, measured using Kendall τ-b (τb) coefficient. We additionally calculated CAGRs for brand-name medication costs within each class. All analyses were performed using Stata statistical software version 15.1 (StataCorp) and Excel spreadsheet software version 14.7.6 (Microsoft). Data were analyzed in August 2020.

## Results

**Figure 1.**
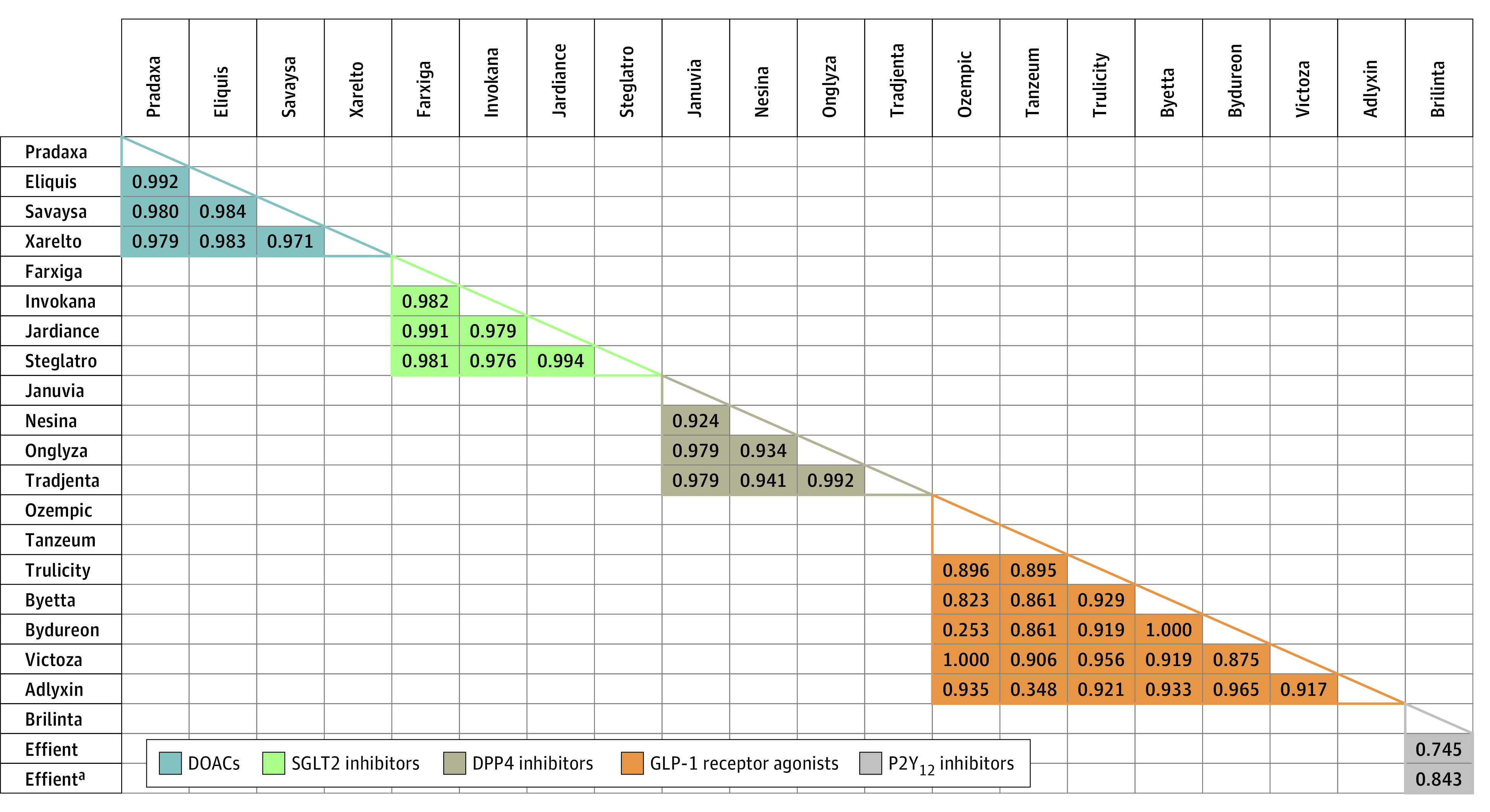
Trends in Kendall τb Coefficients Among Brand-Name Medications Within the Same Drug Class from 2015 to 2020 ^a^A generic form of prasugrel was introduced in August 2017; this value represents the Kendall τb coefficient between Brilinta and Effient prior to August 2017. DOAC indicates direct oral anticoagulant; SGLT2, sodium-glucose cotransporter-2; DPP4, dipeptidyl peptidase-4; and GLP-1, glucagon-like peptide-1.

**Figure 2.**
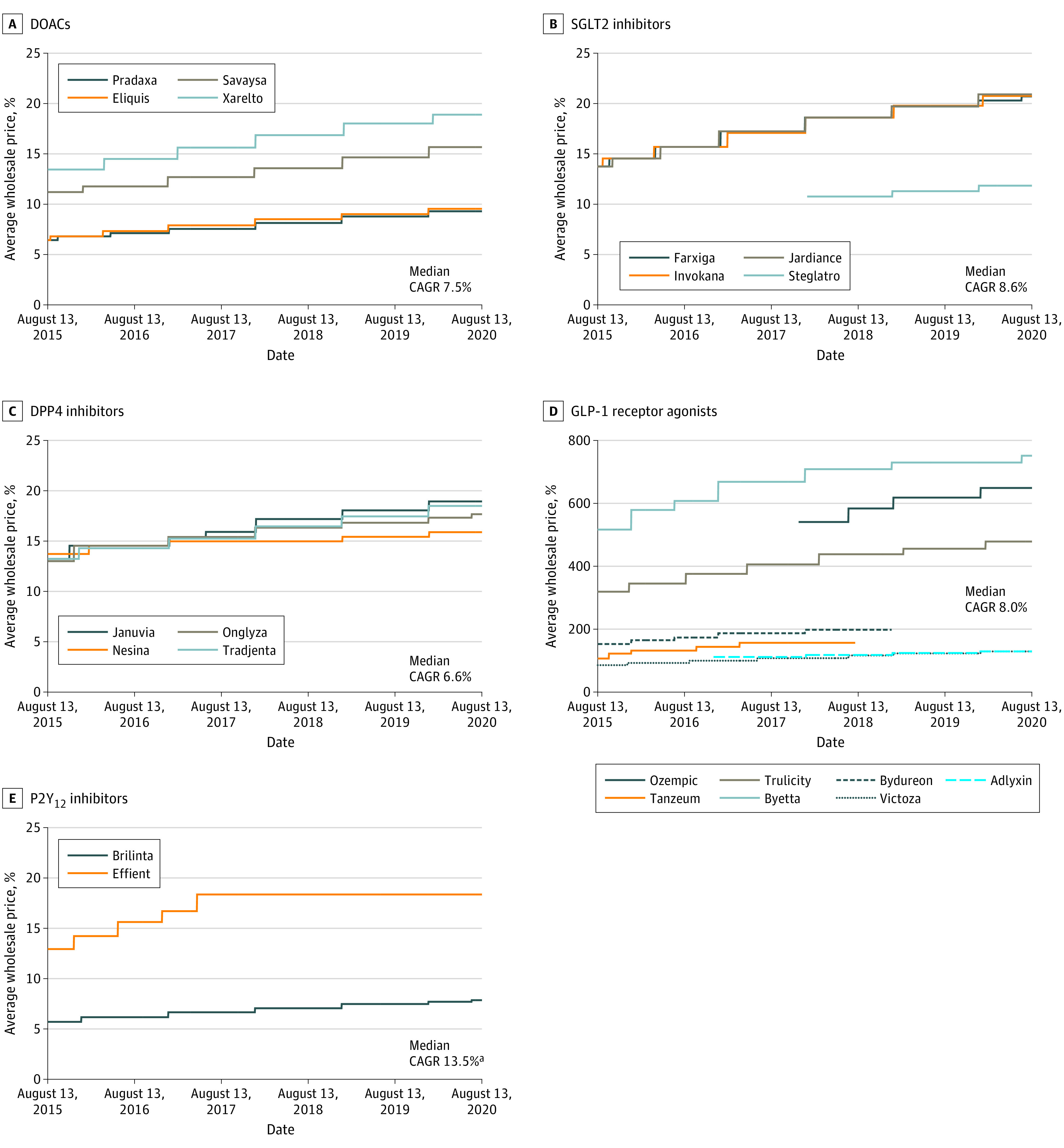
Trends in Average Wholesale Prices Among Brand-Name Medications Within the Same Drug Class from 2015 to 2020 ^a^A generic form of prasugrel was introduced in August 2017; this value represents the median compound annual growth rate (CAGR) of Brilinta and Effient prior to August 2017. DOAC indicates direct oral anticoagulant; SGLT2, sodium-glucose cotransporter-2; DPP4, dipeptidyl peptidase-4; and GLP-1, glucagon-like peptide-1.

This study included 4 DOACs, 4 SGLT2 inhibitors, 4 DPP4 inhibitors, 7 GLP-1 receptor agonists, and 2 P2Y_12_ inhibitors. The median (range) τb values for drugs within each class were 0.98 (0.97-0.99) for DOACs, 0.98 (0.98-0.99) for SGLT2 inhibitors, 0.96 (0.92-0.99) for DPP4 inhibitors, 0.92 (0.25-1.00) for GLP-1 receptor agonists, and 0.75 between P2Y_12_ inhibitors; however the τb value for P2Y_12_ inhibitors was 0.84 when restricted to August 2015 and August 2017, after which generic prasugrel became available (Figure 1). The median (range) CAGRs in costs over this 5-year period ranged from 6.6% (3.0%-7.4%) for the 4 DPP4 inhibitors to 13.5% (8.0%-19.1%) for the 2 P2Y_12_ inhibitors (Figure 2).

## Discussion

This cross-sectional study found high correlations between AWPs among drugs within 5 classes used to treat chronic conditions that had multiple brand-name medications on the market contemporaneously from 2015 to 2020. Moreover, the median CAGR in costs for each of these medication classes outpaced annual growth rate of the consumer price index for prescription drugs at 2.1% over the same time period.^4^ These results suggest there was little price competition among the sponsors of these products. In fact, for 1 class, P2Y_12_ inhibitors, the correlation between rising AWPs was higher when our analyses were restricted to the period prior to the market introduction of a within-class generic equivalent.

There are some limitations to our analysis. For instance, our findings may not generalize to other drug classes. Moreover, we did not investigate competition across drug classes, which may affect within-class price dynamics. In addition, AWPs do not account for rebates, which are negotiated annually. Rebates, list prices, and net prices have been growing for brand-name medications, and rebate growth has been shown to positively correlate with list price growth, thereby impacting costs faced by patients paying a percentage of (or the full) list price.^5,6^ Therefore, the lock-step price increases of brand-name medications, without evidence of price competition, raise concerns and would be expected to adversely affect patient adherence to medications and thus clinical outcomes. Policies that limit lock-step price increases, shorten patent durations, and encourage development of generic equivalents may mitigate rising drug prices.
